# Deep repertoire mining uncovers ultra-broad coronavirus neutralizing antibodies targeting multiple spike epitopes

**DOI:** 10.1016/j.celrep.2024.114307

**Published:** 2024-06-05

**Authors:** Jonathan Hurtado, Thomas F. Rogers, David B. Jaffe, Bruce A. Adams, Sandhya Bangaru, Elijah Garcia, Tazio Capozzola, Terrence Messmer, Pragati Sharma, Ge Song, Nathan Beutler, Wanting He, Katharina Dueker, Rami Musharrafieh, Sarah Burbach, Alina Truong, Michael J.T. Stubbington, Dennis R. Burton, Raiees Andrabi, Andrew B. Ward, Wyatt J. McDonnell, Bryan Briney

**Affiliations:** 1 Department of Immunology and Microbiology, The Scripps Research Institute, La Jolla, CA 92037, USA; 2 Center for Viral Systems Biology, The Scripps Research Institute, La Jolla, CA 92037, USA; 3 Scripps Center for HIV/AIDS Vaccine Development, The Scripps Research Institute, La Jolla, CA 92037, USA; 4 Division of Infectious Diseases, Department of Medicine, University of California, San Diego, La Jolla, CA 92037, USA; 5 10x Genomics, Inc., 6230 Stoneridge Mall Road, Pleasanton, CA 94588, USA; 6 Department of Integrative Structural and Computational Biology, Scripps Research, La Jolla, CA 92037, USA; 7 IAVI Neutralizing Antibody Center, The Scripps Research Institute, La Jolla, CA 92037, USA; 8 Ragon Institute of MGH, Harvard and MIT, Cambridge, MA 02139, USA; 9 Multi-Omics Vaccine Evaluation Consortium, The Scripps Research Institute, La Jolla, CA 92037, USA

## Abstract

The development of vaccines and therapeutics that are broadly effective against known and emergent coronaviruses is an urgent priority. We screened the circulating B cell repertoires of COVID-19 survivors and vaccinees to isolate over 9,000 severe acute respiratory syndrome coronavirus 2 (SARS-CoV-2)-specific monoclonal antibodies (mAbs), providing an expansive view of the SARS-CoV-2-specific Ab repertoire. Among the recovered antibodies was TXG-0078, an N-terminal domain (NTD)-specific neutralizing mAb that recognizes diverse alpha- and beta-coronaviruses. TXG-0078 achieves its exceptional binding breadth while utilizing the same VH1–24 variable gene signature and heavy-chain-dominant binding pattern seen in other NTD-supersite-specific neutralizing Abs with much narrower specificity. We also report CC24.2, a pan-sarbecovirus neutralizing antibody that targets a unique receptor-binding domain (RBD) epitope and shows similar neutralization potency against all tested SARS-CoV-2 variants, including BQ.1.1 and XBB.1.5. A cocktail of TXG-0078 and CC24.2 shows protection *in vivo*, suggesting their potential use in variant-resistant therapeutic Ab cocktails and as templates for pan-coronavirus vaccine design.

## In brief

Broad and potently neutralizing antibodies aid in identifying conserved sites of viral vulnerability; however, such antibodies are often very rare. Hurtado et al. utilize a next-generation antigen barcoding approach to deeply survey the SARS-CoV-2-specific antibody response in COVID-19 survivors and vaccinees, aiming to identify a cocktail of protective antibodies.

## Graphical Abstract

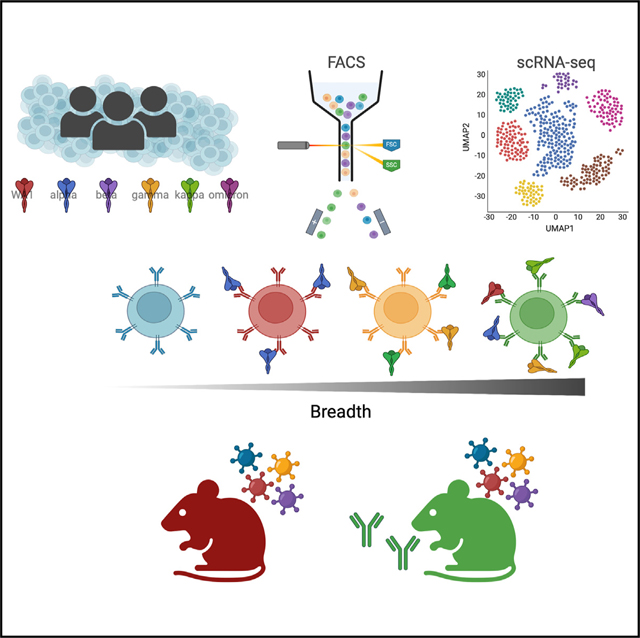

## INTRODUCTION

In the past two decades, three novel coronaviruses (CoVs) have crossed from zoonotic hosts into humans and acquired the ability to spread by human-to-human transmission. Two of these pandemic CoVs, severe acute respiratory syndrome (SARS)-CoV in 2002 and Middle East respiratory syndrome-CoV first identified in 2012, have caused relatively small outbreaks of human respiratory disease.^1,2^ The third, SARS-CoV-2, resulted in a pandemic of CoV disease 2019 (COVID-19) with higher global mortality than any acute virus outbreak in over a century.^3^

Current vaccines provided protection against the ancestral strain of SARS-CoV-2 and are still effective at preventing hospitalization but provide less protection against newly emerged variants.^4^ Furthermore, the ongoing emergence of increasingly diverse variants of concern (VoCs) and the possibility of future spillovers of novel CoVs make pan-CoV vaccine development a public health issue of the highest priority. SARS-CoV-2 infection and vaccination both induce antibody (Ab) and T cell-mediated immunity that is associated with reduced disease severity and transmission.^5,6^ These responses develop in humans as immune ensembles—polyclonal populations of naive and recalled antigen-experienced immune cells, which can target new, previously seen, or evolutionarily related antigens by searching an extremely diverse Ab repertoire space.^7–9^ In response, many pathogens have developed sophisticated immune evasion mechanisms that conceal neutralizing epitopes or distract the Ab response with highly immunogenic, non-neutralizing epitope regions.^10^ Thus, broad and potently neutralizing Abs against such “evasion-strong” viruses are typically quite rare, and their discovery often requires very deep sampling of the pathogen-specific Ab repertoire.^11^ Such Abs are vitally important, however, both as potential clinical treatments and to inform the design of vaccine immunogens that focus the immune response toward conserved regions of viral vulnerability.^12^

Most known monoclonal Abs (mAbs) against SARS-CoV-2 recognize one of three major epitope regions: the receptor-binding domain (RBD), the S2 subunit, and the N-terminal domain (NTD). Neutralizing mAbs (nAbs) have been reported against each of these regions,^13,14^ with the broadest nAbs targeting epitopes in S2^15–19^ or the RBD.^20,21^ In contrast, NTD-specific mAbs primarily target a single supersite using a limited genetic vocabulary^22,23^ and, because the NTD is not particularly well conserved across CoVs, are presumed to be of limited breadth.^24^ Recently reported NTD-specific mAbs targeting a moderately conserved epitope outside the NTD supersite neutralized several SARS-CoV-2 VoCs, but their breadth did not extend beyond SARS-CoV-2 to other human CoVs.^25^

**Figure 1.**
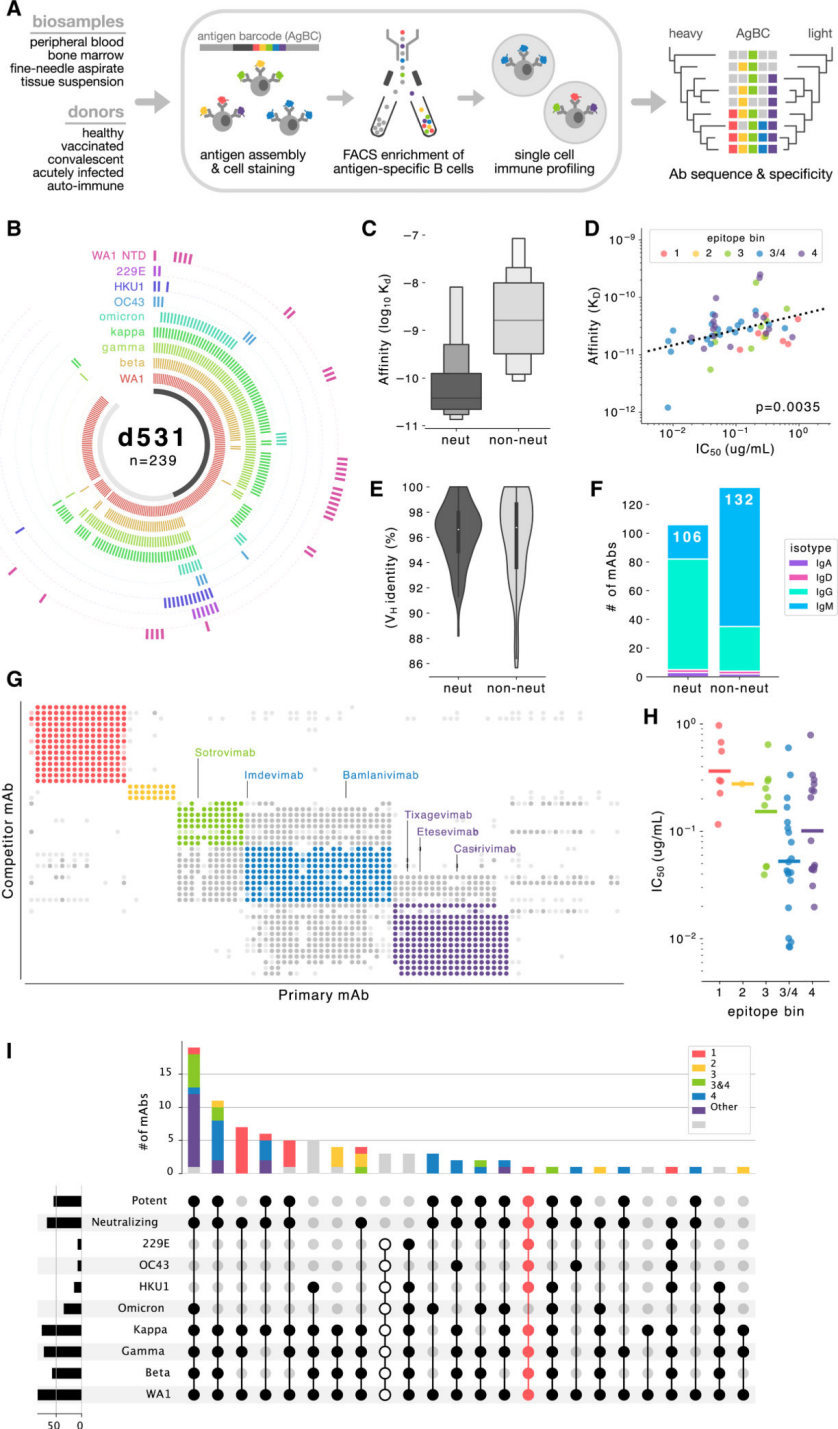
Discovery of an ultra-broad neutralizing antibody targeting the NTD (A) Overview of the multiplexed antigen screening method. (B) Binding specificities of recombinantly expressed mAbs from donor 531 (d531). Each arc segment corresponds to a single mAb, and the ring corresponding to each spike antigen is colored if bound by the mAb. The centermost band is colored dark gray if the mAb neutralizes SARS-CoV-2 and light gray if it is non-neutralizing. (C) Letter-value plot of mAb affinity for WA1 spike comparing *neut* (*n* = 106) versus *non-neut* (*n* = 132) groups. The center line on each box represents the median. (D) Correlation between binding affinity (KD) and neutralization (IC_50_) for the WA1 strain of SARS-CoV-2. The linear regression line is shown, as well as the *p* value indicating a significant positive slope. (E) Violin plot of heavy-chain variable (VH) germline gene identity. 100% identity indicates no somatic mutation. (F) Isotype distribution of WA1 nAbs and non-nAbs. The total number of mAbs in each category (*neut* and *non-neut*) is shown at the top of the respective bar. (G) Epitope binning by competitive binding inhibition. For each mAb pair, a darkly filled circle indicates strong competition, a lightly filled circle indicates weak competition, and no circle indicates no competition. Points are colored according to their respective epitope bin. (H) Neutralization potency against SARS-CoV-2 of nAbs in each epitope bin. Bins are colored as in (G). (I) Upset plot showing cross-reactivity binding and neutralization patterns among epitope-binned mAbs. The broadest cross-reactivity group, which bind all tested CoV S-proteins but do not neutralize, are indicated by unfilled dots. TXG-0078, which comprises its own cross-reactivity pattern, is highlighted in pink.

Here, we employ a multiplexed antigen screening method using single-cell immune profiling technology,^26^ which facilitates the rapid recovery of thousands of natively paired Ab sequences together with detailed antigen specificity information (Figure 1A). We applied this method to a single convalescent COVID-19 survivor and to a small cohort of longitudinally sampled COVID-19 patients who were infected early in the pandemic and subsequently vaccinated, yielding more than 9,000 SARS-CoV-2-specific monoclonal Abs (mAbs). This comprehensive survey enables the targeted discovery of extremely rare but desirable Ab specificities while simultaneously providing an expansive view of the pathogen-specific Ab repertoire. The rich single-cell profiles generated with this workflow allow fine-grained deconvolution of Ab binding patterns, revealing that increased Ab breadth following vaccination of previously infected individuals is due to the broadening of individual mAbs rather than supplementation by narrow but complementary mAb specificities. We observed substantial clonal convergence among SARS-CoV-2-specific mAbs, in which mAbs from different individuals coalesce upon similar “public” genetic solutions to pathogen recognition. We recovered two particularly interesting nAbs: CC24.2, which targets a novel RBD epitope and exhibits pan-sarbecovirus breadth that includes the BA, BQ, and XBB variants of the SARS-CoV-2 Omicron lineage, and TXG-0078, an NTD-specific nAb able to recognize a diverse range of alpha- and beta-CoVs. TXG-0078 alone, as well as a cocktail of TXG-0078 and CC24.2, protects against *in vivo* SARS-CoV-2 challenge when given prophylactically. Broadly protective mAb cocktails are in some ways preferable to monotherapy, as increased epitope diversity provides added protection against viral escape. The discovery of these novel conserved neutralizing epitopes will also inform the future development of pan-CoV vaccines.

## RESULTS

We first obtained approximately 10^8^ peripheral blood mononuclear cells from a convalescent participant with prior PCR-confirmed SARS-CoV-2 infection. Using a small, multiplexed antigen bait panel consisting of soluble prefusion trimeric WA1 spike (S-protein), prefusion trimeric D614G S-protein, and human serum albumin as a negative control, antigen-specific B cells were enriched by flow cytometric sorting and processed using the 10× Genomics Chromium platform, recovering 2,737 natively paired mAbs.

We selected a subset of 239 mAbs for recombinant expression and detailed characterization (Figure 1B). Regardless of the original isotype, all mAb variable regions were recombinantly expressed using an immunoglobulin G1 (IgG1) backbone vector. Most mAbs detectably bound SARS-CoV-2 (197 of 239; 82%), demonstrating the high purity with which this workflow enriches antigen-specific B cells. It is important to note that this binding fraction likely underestimates the selectivity of this method. Non-binding mAbs were overwhelmingly IgM (34 of 42; 81%), and it is possible that some, perhaps many, of these mAbs rely more heavily on avidity for S-protein binding. Avidity-driven interactions would likely be overlooked when evaluating these mAbs as recombinant IgGs. Binding mAbs were generally of high affinity, with 130 of 197 (66%) mAbs producing apparent K_D_ values in the picomolar range.

Of the 239 recombinantly expressed mAbs, 106 (44%) neutralized the WA1 strain of SARS-CoV-2 in a live virus red dye uptake assay. As a group, neutralizing mAbs (nAbs) bound soluble WA1 S-protein with higher affinity than did non-nAbs (Figure 1C). Binding affinity correlated with neutralization potency (Figure 1D), indicating that low affinity may be at least partially responsible for the lack of detectable neutralization by non-nAbs. Although nAbs and non-nAbs showed similar levels of somatic mutation (Figure 1E), nAbs were predominantly IgG (73%), while a similarly sized majority of non-nAbs were IgM (73%; Figure 1F). This parallels the results seen with non-binding mAbs and again suggests that avidity-reliant IgM mAbs may not reproduce their native function when expressed recombinantly as IgGs. Epitope binning by competitive binding inhibition identified five major specificity groups (Figure 1G). Three groups (3, 3/4, and 4) represent the RBD epitopes targeted by marketed therapeutic mAbs, and the remaining two groups (1 and 2) correspond to distinct epitope regions on the NTD. Neutralizing mAbs against RBD epitopes were generally more potent than NTD nAbs, with the most potent nAbs belonging to competition bin 3/4 (Figure 1H).

Screening against a diverse panel of CoV S-proteins, including several SARS-CoV-2 VoCs and seasonal CoVs, revealed a variety of cross-reactivity binding patterns. The Beta S-protein escaped recognition by most mAbs in epitope bin 1, and very few NTD mAbs (epitope bins 1 and 2) were able to bind the Omicron S-protein. To assess cross-reactivity, we grouped the mAbs by their ability to neutralize WA1 and bind to a variety of CoV S-proteins. The largest group contained mAbs that neutralize WA1 and bind the S-proteins of all tested SARS-CoV-2 VoCs, including Omicron (Figure 1I). This is particularly notable because the donor sample was collected over a year before the Omicron variant was first detected. The only mAbs able to recognize all SARS-CoV-2 VoCs and seasonal CoV S-proteins were non-neutralizing, although one especially interesting NTD-specific mAb, named TXG-0078 and highlighted in pink in Figure 1I, bound all tested isolates except Omicron and potently neutralized SARS-CoV-2. TXG-0078 uses the IGHV1–24 germline gene segment, which is characteristic of mAbs targeting the NTD supersite.^22,25^ Despite this similarity to existing mAbs, TXG-0078 is the only known neutralizing mAb against the NTD supersite with binding breadth that spans alpha- and beta-CoVs.

### Development of Ab breadth following infection and vaccination

**Figure 2.**
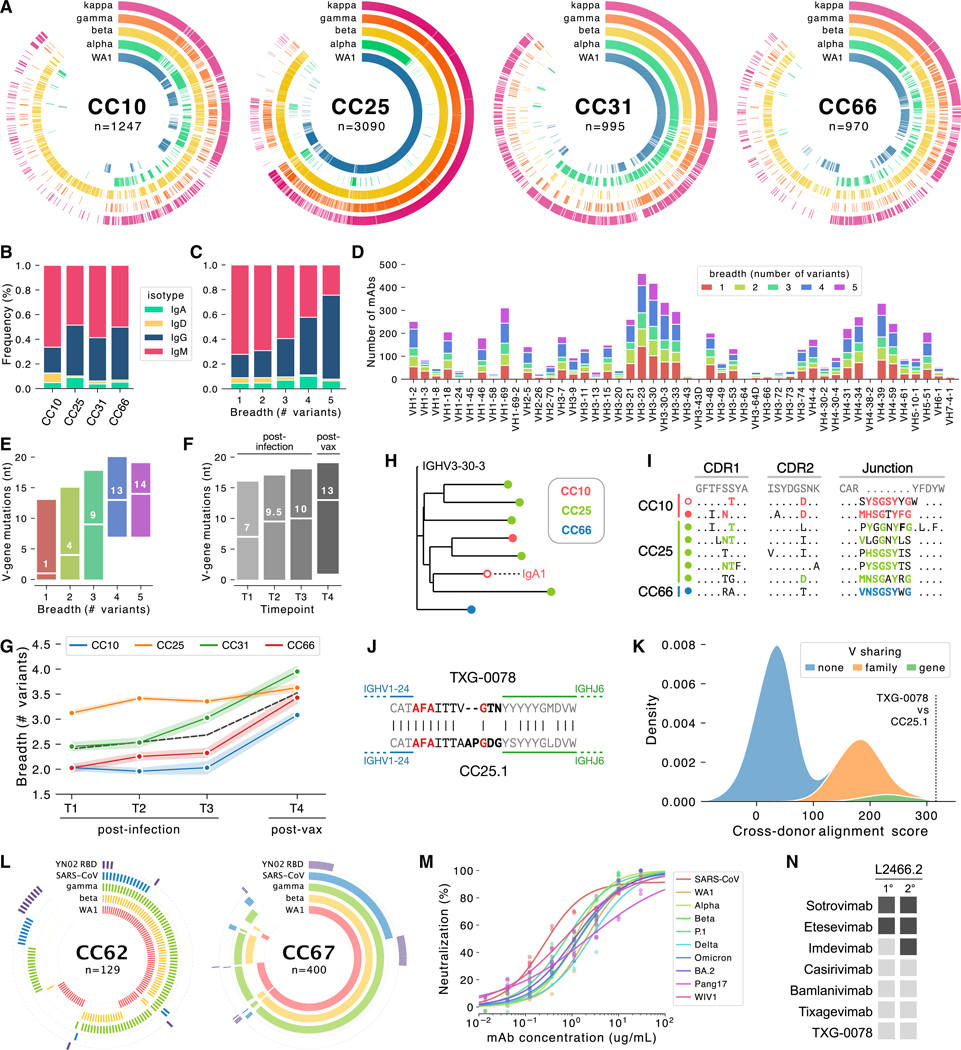
Quantifying the development of mAb breadth of binding using antigen barcodes (A) Ring plots showing the antigen barcode classifications of 6,305 SARS-CoV-2-specific mAbs from participants CC10, CC25, CC31, and CC66 (additional participants found in Figure S1). Individual mAbs are represented by radial vectors, with each antigen ring (WA1: blue, Alpha: green, Beta: yellow, Gamma: orange, Kappa: magenta) colored if the given B cell was classified as positive for the respective antigen. Antibodies are sorted by breadth, with breadth decreasing in the clockwise direction. (B) Isotype distribution of SARS-CoV-2-specific mAbs for each donor. (C) Isotype distribution of mAbs, grouped by cross-variant breadth. (D) VH gene use of SARS-CoV-2-specific mAbs, grouped by cross-variant breadth. (E) Number of VH nucleotide mutations in SARS-CoV-2-specific mAbs, grouped by breadth. White line represents the median, with the inter-quartile interval representing 50% of the data. (F) Number of VH nucleotide mutations in SARS-CoV-2-specific mAbs, grouped by time point (T1, T2, and T3 are post-infection but pre-vaccination and T4 is post-infection and post-vaccination). (G) Median cross-variant breadth of all mAbs isolated at each time point, grouped by donor. The dashed black line represents the median of all mAbs from participants. (H) Phylogenetic representation of a single public SARS-CoV-2-specific clonotype. All mAbs in the clonotype use the IgG1 isotype except for a single IgA1 clone (indicated by text and an unfilled marker). (I) Complementarity-determining region (CDR) alignments of sequences in the public clonotype shown in (H). Mutations (CDR1 and −2) or untemplated residues (junction) shared between multiple participants are highlighted in color (CC10: magenta, CC25: green, CC66: blue). The clone IgA1 mAb is indicated by an unfilled marker. (J) Junction alignments of a second public clonotype comprising TXG-0078 (from donor 531) and CC25.1 (from donor CC25). Untemplated positions (N- and P-additions) are indicated in bold, and identical residues in untemplated regions are highlighted in red. (K) Distribution of cross-donor alignment scores obtained by iterative pairwise alignment of heavy-chain amino acid sequences. Only sequences recovered from different doors were compared. Distributions resulting from a comparison of sequences encoding the same V-gene (green), V-genes from the same family (orange) or V-genes from different families (blue) are shown. The alignment score between TXG-0078 and CC25.1 is highlighted. (L) Ring plots showing antigen barcode classifications of two additional participants (CC62 and CC67). Antigen rings are colored if the respective mAb was classified as antigen positive (WA1: magenta, Beta: yellow, Kappa: green, SARS-CoV: blue, YN02 RBD: purple). Ring plots for two additional donors (CC24 and CC42) can be found in Figure S1. (M) Neutralization of SARS-CoV (red) and several SARS-CoV-2 variants by mAb CC24.2. Neutralization assays were performed in triplicate at each concentration. (N) Epitope binning of mAb CC24.2 with several RBD-specific mAbs. Two competitor mAbs (CC25.4 and CC25.56) bind the site 5 epitope, and the other competitor mAbs (CC25.54, CC84.24, and CC84.2) bind other RBD epitopes. Binding inhibition (dark gray) or lack of binding inhibition (light gray) is shown, with PBS used as a negative control.

We next assessed the SARS-CoV-2-specific Ab response using a larger multiplexed antigen panel designed to quantify cross-VoC binding breadth. Using a longitudinal cohort of participants who had been infected with SARS-CoV-2 (post-infection time points T1, T2, and T3) and subsequently vaccinated (post-vaccination time point T4), we isolated a total of 6,302 paired mAbs (Figure 2A). The isotype distribution was similar across all four participants, and although the majority of SARS-CoV-2-specific mAbs were IgM (58%, Figure 2B), the use of IgG increased markedly with breadth (18.3% in single-variant binders, 65.6% in pan-VoC binders; Figure 2C). Heavy-chain variable gene (V-gene) use among SARS-CoV-2-specific mAbs was quite diverse, and we did not observe substantial enrichment of specific V-genes among cross-reactive mAbs (Figure 2D). Broadly reactive mAbs were more somatically mutated than single-variant binders (Figure 2E), which, together with the isotype composition observations, suggests an association between increased maturation (class-switching and somatic hypermutation) and binding breadth. Interestingly, although somatic mutation frequency increased consistently over time (Figure 2F), mAb breadth remained relatively static following infection before substantially increasing after vaccination for three of four participants (Figure 2G).

### Frequent clonal coalescence among SARS-CoV-2 Abs

To quantify the occurrence of “public” CoV-specific mAbs, which are highly similar mAbs present in multiple different individuals, we performed pairwise comparisons of every possible combination of CoV-specific mAbs from different participants and ranked them by similarity. Sequences from one such group of public mAbs, isolated from three different participants, showed maturation trajectories of sufficient similarity that cross-donor sequences were phylogenetically interspersed (Figure 2H). Indeed, the similarity between these public Ab sequences was not attributable solely to the use of identical germline gene segments but was also the result of substantial homology in non-germline encoded residues (Figure 2I). We refer to this phenomenon—sequences with similar germline rearrangements that also follow similar maturation trajectories—as clonal coalescence. We also observed clonal coalescence between TXG-0078, the exceptionally broad NTD-specific nAb described above, and another CoV S-specific mAb, CC25.1, which was isolated from the post-vaccination sample of donor CC25 (Figures 2A and 2J). Like the coalescence group shown in Figures 2H and 2I, the heavy-chain junctions of TXG-0078 and CC25.1 were remarkably similar even at non-templated positions, resulting in much greater similarity than would be expected by merely encoding the same germline components (Figure 2K). TXG-0078 and CC25.1 were isolated at different facilities that did not share samples or reagents, eliminating the possibility of contamination as a cause of the observed clonal coalescence.

### Isolation of a pan-sarbecovirus RBD nAb using an expanded antigen panel

Encouraged by the increased breadth observed in participants who had been both infected and vaccinated, we subsequently screened additional participants using a modified antigen panel designed to assess the breadth of binding beyond SARS-CoV-2 variants. This panel included S-proteins from the WA1, Beta, and Gamma strains of SARS-CoV-2, as well as SARS-CoV S-protein and the RBD from YN02, a clade 2 bat CoV closely related to SARS-CoV-2 (Figure 2L). We observed a surprisingly high frequency of mAbs that bound both SARS-CoV-2 and SARS-CoV (10%–20% of all mAbs). One particularly broad mAb, named CC24.2, neutralized SARS-CoV and a broad range of SARS-CoV-2 variants, including Omicron, BA.2, BQ.1.1, and XBB.1.5 (Figure 2M). The similar potency with which CC24.2 neutralized all tested sarbecovirus isolates suggests either high conservation of critical contact residues or sufficient binding flexibility that some epitope variability can be tolerated. We also note the near-perfect functional complementarity between TXG-0078 and CC24.2: TXG-0078 binds a broad range of CoVs but cannot recognize SARS-CoV or Omicron variants of SARS-CoV-2, while CC24.2 does not exhibit breadth beyond sarbecoviruses but neutralizes SARS-CoV and all tested Omicron variants. Epitope mapping of CC24.2 by competitive binding indicates a specificity that overlaps two mAbs against a novel RBD epitope (R.A. and I. Wilson, unpublished data), which has been termed site 5 (Figure 2N).

### Structural definition of a broadly conserved NTD neutralizing epitope

**Figure 3.**
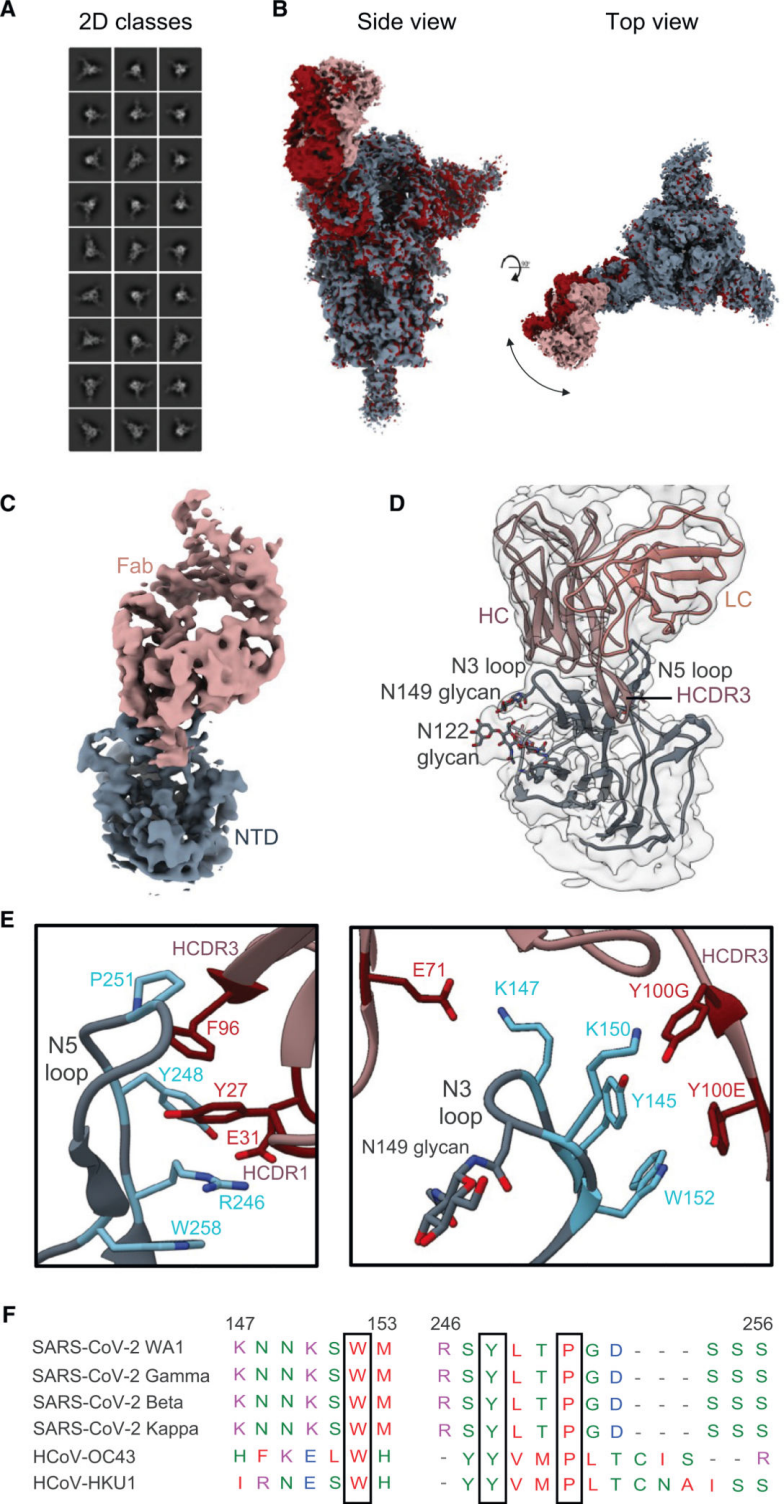
Structural definition of the broad, NTD-supersite antibody TXG-0078 (A) Representative 2D classes obtained by cryoelectron microscopy (cryo-EM) analysis of TXG-0078 Fab in complex with SARS-CoV-2 spike. (B) Two cryo-EM reconstructions of the TXG-0078-spike complex at 3.14 (rosy brown/gray) and 3.34 Å (red) resolutions showing conformational flexibility of the Fab-NTD region. (C) Cryo-EM reconstruction (3.9 Å) of the NTD-Fab region obtained by local refinement. The spike NTD is colored gray, and the Fab is colored rosy brown. (D) The atomic model of TXG-0078 Fab Fv bound to NTD docked in the local refinement map. (E) The atomic model reveals that TXG-0078 targets the NTD supersite, as has been described for other antibodies, though it is capable of weakly binding endemic human coronaviruses. The N3 and N5 loops comprise the epitope of TXG-0078; the CDRH3 of TXG-0078 reaches into a pocket in the N5 loop, and the light chain is only minimally engaged in binding. Epitope and paratope residues are colored in light blue and red, respectively. (F) Multiple sequence alignment of spikes from diverse TXG-0078-reactive beta-CoVs was performed using Clustal Omega. Epitope residues are displayed in (F), showing conservation of proline at position 251.

Although many NTD-specific nAbs have been reported, none have demonstrated the binding breadth of TXG-0078, making the specific molecular interactions between TXG-0078 and S-protein of particular interest. Our cryoelectron microscopy analysis of TXG-0078 in complex with the WA1 SARS-CoV-2 S-protein resulted in 2 different reconstructions of the spike-Fab complex (3.14 and 3.34 Å), displaying the conformational flexibility of the Fab-NTD region (Figures 3A, 3B, and S2; Table S1). We further performed local refinement in the NTD-Fab region, which yielded a 3.9 Å reconstruction (Figure 3C). XG-0078 binds the N3 and N5 loops of the NTD using all three heavy-chain complementarity-determining region (HCDR) loops (Figure 3D). Several aspects of TXG-0078 binding are similar to 4A8, a previously reported NTD-specific nAb.^14^ Both mAbs use a similar angle of approach, encode the same heavy-chain V-gene, and share binding interactions between the NTD and their HCDR1 and HCDR2 loops. The shared interactions include R246 contacts with HCDR1 residues Y27 and E31, an H146 H-bond with T30 on HCDR1, salt bridge formation between K147 and residue E71 on HCDR2, and a K150 H-bond with Y100^G^ on HCDR3. However, the HCDR3s of the two mAbs differ at multiple residues, allowing TXG-0078 to make additional contacts not observed with 4A8 (Figure 3E). Notably, TXG-0078 has a phenylalanine at position 96 on the HCDR3 instead of a threonine in 4A8, resulting in CH/*π* interactions with P251 on the NTD while also forming stabilizing contacts with Y27 on HCDR1. The proline at position 251 is conserved across all TXG-0078-reactive CoV spikes, WA1, Beta, Gamma, Kappa, OC43, and HKU1, allowing TXG-0078 to accommodate variability at other contact positions in a way that less broad NTD-specific mAbs cannot (Figure 3F).

### Ab-mediated protection against SARS-CoV-2 infection

**Figure 4.**
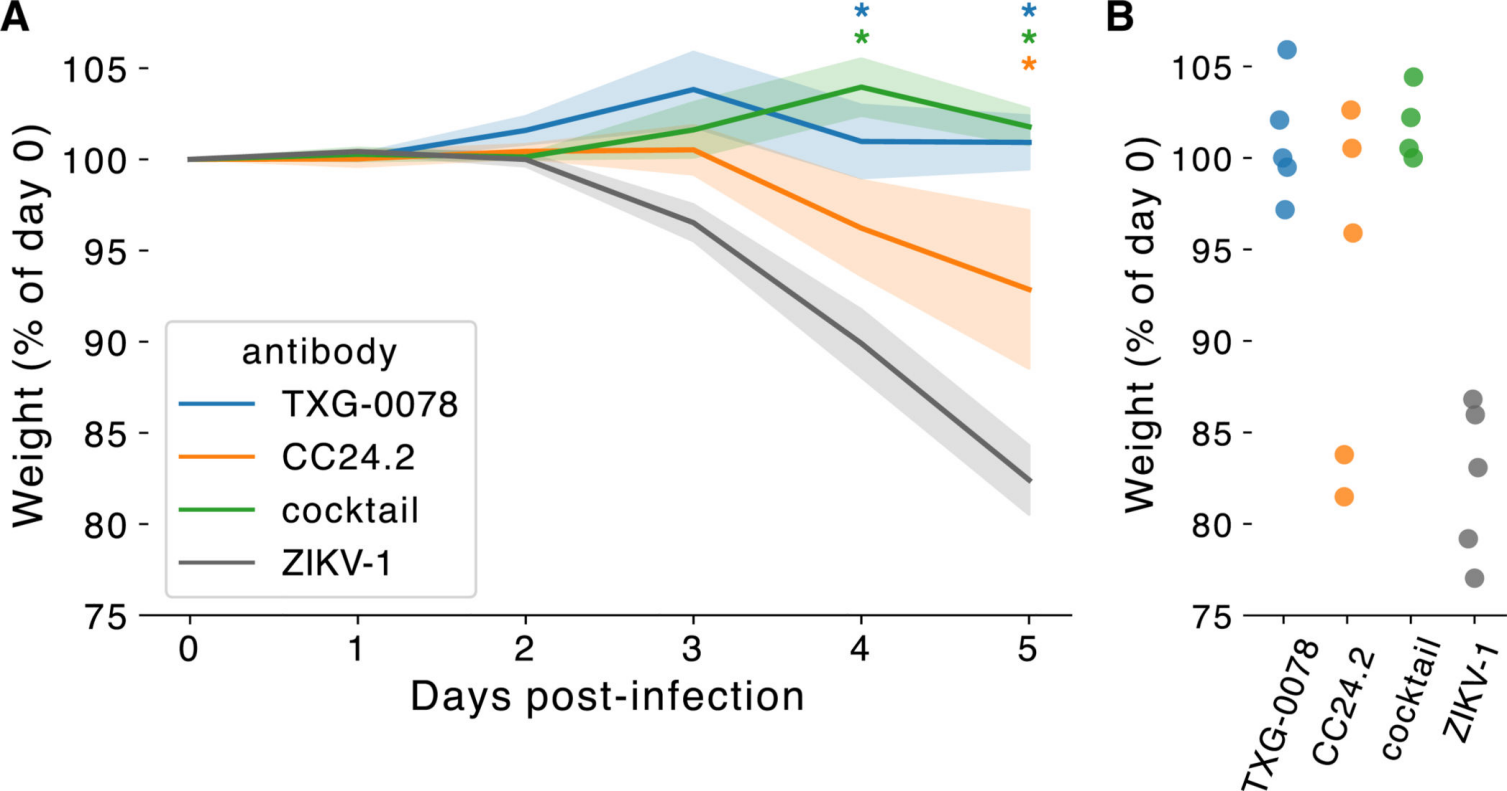
TXG-0078 protects transgenic ACE2 mice from live SARS-CoV-2 challenge (A) Mice (*n* = 20) were administered 300 μg of TXG-0078, CC24.2, a cocktail of both TXG-0078 and CC24.2, or a control antibody targeting Zika virus (ZIKV-1). Each group contains 5 animals. The mean percentage of change in baseline weight is shown for each group. Shading indicates standard error. Significant differences between groups (*p* ≤ 0.05) are indicated by asterisks in the color of each group displaying significantly different weight loss than the control (ZIKV-1) group. Significance was determined using a one-way analysis of variance (ANOVA) with Dunnet’s multiple comparisons test. (B) Percentage of baseline weight at day 5 is shown for all mice, separated by treatment group.

As described above, TXG-0078 neutralizes SARS-CoV-2 and binds a diverse array of alpha- and beta-CoVs including OC43, HKU1, and 229E; however, TXG-0078 was unable to neutralize SARS-CoV or Omicron variants of SARS-CoV-2. CC24.2 neutralizes all tested sarbecoviruses, including SARS-CoV and Omicron variants of SARS-CoV-2. Due to their complementary specificities, we evaluated the potential of TXG-0078 and CC24.2 to protect against *in vivo* SARS-Cov-2 challenge alone or in combination. We administered 300 μg of prophylactic mAb to a total of 20 ACE2 transgenic mice in four treatment groups: TXG-0078, CC24.2, a two-mAb cocktail comprising 150 μg each of TXG-0078 and CC24.2, and ZIKV-1, a negative control Ab targeting Zika virus. Following mAb administration, mice were challenged with the WA1 strain of SARS-CoV-2 through intranasal administration. Each mouse was weighed at day 0 (baseline) and again daily for 5 days before sacrifice. All mice receiving TXG-0078 or the cocktail were protected from weight loss (Figure 4). CC24.2 appeared to provide partial protection, with 3 of 5 mice exhibiting a reduction in weight loss comparable to animals receiving TXG-0078 or the cocktail. The reduced efficacy of CC24.2 can likely be attributed to its approximately 10-fold lower potency against SARS-CoV-2 compared to TXG-0078 (IC_50_ values of 1.63 μg/mL and 171 ng/mL, respectively).

## DISCUSSION

As the COVID-19 pandemic made painfully clear, the development of broad, variant-resistant vaccines and therapeutics is urgently necessary to prevent and control future outbreaks of infectious diseases with pandemic potential. Integral to rational vaccine and therapeutics discovery strategies is the identification of conserved regions of viral vulnerability. Unfortunately, conserved neutralizing epitopes are often cryptic or poorly immunogenic, and potent Abs targeting such regions can be extremely rare. Here, we demonstrate the power of multiplexed antigen screening using single-cell immune profiling technology for deeply surveying and cataloging functional adaptive immune repertoires by isolating over 9,000 CoV-specific human mAbs. This expansive mAb dataset revealed multiple instances of cross-reactivity among the tested VoCs. Notably, donor 531 had Omicron cross-reactive Abs prior to the Omicron wave, highlighting the high structural similarity between these VoCs. Adding to the intrigue, there were multiple occurrences of clonal coalescence, in which different participants independently produced antigen-specific mAbs with remarkable genetic similarity. Of particular interest was TXG-0078, a public NTD-specific neutralizing mAb that binds a broad range of alpha- and beta-CoVs and which provided protection against *in vivo* challenge with SARS-CoV-2. The discovery of a conserved neutralizing NTD epitope has exciting implications for the development of therapeutic mAb cocktails that improve resistance to viral escape by targeting multiple conserved epitopes, as well as the development of vaccines that protect against current, emerging, and future human CoVs. We also report the discovery of CC24.2, a neutralizing mAb that achieves pan-sarbecovirus breadth by targeting a novel RBD epitope.

Beyond SARS-CoV-2, the ability to perform large-scale functional analyses of circulating B cells allows much more sophisticated and granular analysis of the pathogen-specific Ab response than can be achieved using traditional serology or mAb isolation techniques. With serology, for example, it is not possible to determine whether broadly reactive serum is the result of broadly reactive mAbs or a complementary combination of narrow specificities. Using this single-cell multiplexed antigen screening approach, we can interrogate the genetics and binding specificities of thousands of B cells simultaneously, producing an expansive representation of the pathogen-specific humoral response at single mAb resolution. This approach has the potential to greatly advance our understanding of infectious disease biology; speed the discovery of exceptionally rare mAbs with unique, therapeutically desirable specificities; and facilitate the design and evaluation of vaccines that elicit durable, variant-resistant immunity.

### Limitations of the study

The primary constraint of the study lies in the translatability of the mouse experiment. The use of only WA1 VoC for challenge does not directly demonstrate the neutralizing breadth of TXG-0078 and CC24.2. However, it does yield promising outcomes that warrant further investigation against a broader panel of VoCs in subsequent studies. Furthermore, the 10-fold lower potency between the two Abs is not optimal in a cocktail framework. Further development of CC24.2 holds promise in enhancing its efficacy. Mouse models come with several limitations that may not translate, in terms of human therapeutic efficacy, yet are invaluable in therapeutic discovery and vaccine research.

## STAR★METHODS

### RESOURCE AVAILABILITY

#### Lead contact

Requests for resources and reagents should be directed to the Lead Contact, Bryan Briney (briney@scripps.edu).

#### Materials availability

All unique/stable reagents generated in this study are available from the lead contact with a completed Materials Transfer Agreement.

#### Data and code availability

All source data are available as part of this publication. Ab sequences, together with single cell gene counts matrices and antigen barcode and cell hash counts data, are available via Zenodo (https://doi.org/10.5281/ZENODO.8432479). CryoEM structures are available via the PDB (8SWH) and EMDB (EMD-40812, EMD-40813, and EMD-40814). Code to replicate key findings of this publication is freely available via GitHub (github.com/brineylab/SARS2-repertoire-mining-paper) under the permissive MIT license.

### EXPERIMENTAL MODEL AND STUDY PARTICIPANT DETAILS

#### Human samples

De-identified peripheral blood mononuclear cells (PBMC) from SARS-CoV2 positive individuals (*n* = 11) were generously supplied from the “Collection of Biospecimens from Persons Under Investigation for 2019-Novel Coronavirus Infection to Understand Viral Shedding and Immune Response Study” (UCSD IRB# 200236), with approval from the UCSD Human Research Protection Program or from commercial procurement.

#### Mouse challenge experiment

K18-hACE2 transgenic mice (The Jackson Laboratory) were used in all challenge experiments. Each group contained *n* = 5 mice. The research protocol was approved and performed in accordance with Scripps Research IACUC Protocol #20–0003.

### METHOD DETAILS

#### Recombinant antibody vector synthesis, transient expression, and purification

Variable heavy chain and light chain domains of anti-SARS-CoV-2 S1 antibodies were reformatted to IgG1 and synthesized and cloned into mammalian expression vector pTwist CMV BG WPRE *Neo* utilizing the Twist Bioscience eCommerce portal. Light chain variable domains were reformatted into kappa and lambda frameworks accordingly. Clonal genes were delivered as purified plasmid DNA ready for transient transfection in HEK Expi293 cells (Thermo Scientific). Cultures in a volume of 1.2 mL were grown for four days, harvested and purified using Protein A resin (PhyNexus) on the Hamilton Microlab STAR platform into 43 mM Citrate 148 mM HEPES, pH 6.

#### Carterra LSA surface plasmon resonance experiments

Surface plasmon resonance experiments were performed on a Carterra LSA SPR biosensor equipped with a HC30M chip at 25°C in HBS-TE (10 mM HEPES pH 7.4, 150 mM NaCl, 3 mM EDTA, 0.05% Tween 20). Antibodies were diluted to 10 μg/mL and aminecoupled to the sensor chip by EDC/NHS activation, followed by ethanolamine HCl quenching. Increasing concentrations of ligand were flowed over the sensor chip in HBSTE with 0.5 mg/mL BSA with 5-min association and 15-min dissociation. Following each injection cycle the surface was regenerated with 2 × 30 s injections of IgG elution buffer (Thermo). Data were analyzed in Carterra’s Kinetics Tool software with 1:1 binding model. Pre-fusion trimeric spike proteins used in the screen included SARS-CoV-2 variants WA-1/2020, beta, gamma, kappa, and omicron, and endemic coronaviruses HKU1, OC-43, and 229E. All antigens were sourced from Acro Biosystems. These reagents contain His tag and T4 foldon domains for purification and trimerization respectively.

#### Vero E6 neutralization assays

Assays were performed with a clinical isolate of the SARS-CoV-2 B.1 lineage (MEX-BC2/2020). This virus carries the D614G mutation in the spike protein (full sequence available at the GISAID/EpiCoV database ID: EPI_ISL_747242). The screen was performed with a microneutralization assay that utilizes prevention of the virus-induced cytopathic effect (CPE) in Vero E6 cells. All antibodies were stored at 4°C until its use. The screen was performed in ten different experiments performed over ten days, each one assessing the activity of approximately 30 antibodies in parallel. All plates included a positive control–plasma from a convalescent patient who had also received the first dose of the Pfizer/BioNTech mRNA vaccine (BNT162b2). Plasma was collected 21 days after the vaccine injection. We utilized Vero E6 cells to evaluate neutralization activity against a replication competent SARS-CoV-2 virus. Antibodies were pre-incubated first with the virus for 1h at 37°C before addition to cells. Following pre-incubation of Ab/virus samples, Vero E6 cells were challenged with the mixture. After addition to cells, antibodies were present in the cell culture for the duration of the infection (96 h), at which time a neutral red uptake assay was performed to determine the extent of the virus-induced CPE. Prevention of the CPE was used as a surrogate marker to determine the neutralization activity of the test-items against SARS-CoV-2. Eight dilutions of each antibody were tested in duplicates for the neutralization assay using a 5-fold dilution scheme starting at 1,000 ng/mL 50% inhibitory concentration (**IC**_**50**_) values of the antibodies displaying neutralizing activity were determined using GraphPad Prism software. Vaccinated plasma control was assessed on each plate using singlet data-points (8 2-fold dilutions throughout 1:20,480). Neutralization data can be found in the supplementary datasets available via Zenodo.

#### Cryo-EM analysis

The design and expression of SARS-CoV-2 HP-GSAS Mut7 spike used for cryo-EM studies was done as previously described.^33^ The spike protein was incubated with 3-fold molar excess of TXG-0078 Fab (final concentration of 1 mg/ml) for 30 min and mixed with 0.5 μL of 0.04 mM Lauryl maltose neopentyl glycol (LMNG) solution immediately prior to sample deposition onto plasma-cleaned Quantifoil 1.2/1.3 grids. Grids were then blotted for 4 s before being plunged into liquid ethane using a Vitrobot mark IV (Thermo Fisher Scientific). Data collection was performed on a Thermo Fisher Glacios operating at 200 keV mounted with a Thermo Fisher Falcon 4 direct electron detector using the Thermo Fisher EPU 2 software. A total of 4250 micrographs were collected at a total dose of 50 e–/Å2. CryoSPARC Live Patch Motion Correction was used for alignment and dose weighting of movies. CTF estimations, particle picking, particle extraction and iterative rounds of 2D classification were performed on CryoSPARC.^27^ The particles were transferred to Relion 3.1 for further processing.^28^ Focused classification involving iterative rounds of alignment-free 3D classification with masks around the NTD-Fab region and 3D refinement was used to obtain two reconstructions of the spike-Fab complex. The local refinement of the NTD-Fab region was performed on cryoSPARC with the local refinement job.

#### Model building and refinement

Initial model building into the local refinement reconstruction was performed manually in Coot using PDB: 7C2L (spike complexed with 4A8 Fab) as a template.^34^ Rosetta relaxed refinement was performed on the initial model and EMRinger and MolProbity metrics were calculated following Rosetta refinement to evaluate and identify the best refined models and Phenix comprehensive validation was performed on the final model.^27,28,32^

#### Animal challenge study

Each group (*n* = 5) was passively immunized with 300μg of each mAb through intraperitoneal injections 24 h prior to infection. Mice were infected with 30,000 FFU of SARS-CoV-2 (WA1/2020). Mice were weighed daily, and percent baseline weight was calculated relative to day 0 weight. On day 5, mice were euthanized and lung lobes were extracted for viral titer quantification.

#### Single cell multiplexed antigen screening workflow

Approximately 4.4 million enriched B cells (selected magnetically from 100 million PBMCs) were resuspended in labeling buffer (1% BSA in PBS) and underwent Fc blocking for 10 min on ice using Human TruStain FcX (BioLegend). Next, cells were stained with the following cocktail of antibodies, antigens and dyes:

CD19 PE-Cy7 (clone SJ25C1, BD Pharmingen) for discrimination of CD19^+^ cells by using fluorescence-activated cell sorting (FACS).

The final conjugated antigens include TotalSeq-C0951 PE trimerized S (SARS-2), TotalSeq-C0952 PE Human Serum Albumin, TotalSeq-C0956 APC trimerized S D614G (SARS-2), TotalSeq-C0957 APC Human Serum Albumin, and 7AAD for live/dead cell discrimination. Cells were stained in labeling buffer (1% BSA in PBS) in the dark for 30 min on ice, then cells were washed 3 times with 2 mL of cold labeling buffer at 350*g for 5 min at 4°C, resuspended in cold labeling buffer and a 1:200 addition of live/dead cell discriminating agent 7AAD for 10 min on ice in the dark, then washed one more time with labeling buffer at 350*g for 5 min at 4°C, then resuspended in labeling buffer and loaded into a Sony MA900 Cell Sorter using a 70 μM sorting chip. Cells were initially gated on being single, live (7AAD-negative) and PE-Cy7-CD19^+^ and then sorted on their PE and/or APC status directly into master mixed and water based on one of four criteria.

1. **PE+**, representing trimerized S (SARS-2) antigen and/or HSA-binding memory B cells.
2. **APC+**, representing trimerized S D614G (SARS-2) antigen and/or HSA-binding memory B cells.
3. **PE+ and APC+**, representing a combination of SARS-2, D614G and/or HSA-binding memory B cells.
4. **PE and APC negative**, representing cells not binding either SARS-2 antigen or HSA.

The resulting volume was adjusted with additional water to match the recommended volume and target for loading with the 10 × 5′V2 Single Cell Immune Profiling kit. Flow cytometry data were analyzed using FlowJo. Standard gene expression, V(D)J, and barcoded antigen libraries were constructed using the 10 × 5′V2 Single Cell Immune Profiling kit per manufacturer’s instructions. Additional information in this regard can be found at https://www.10xgenomics.com/products/single-cell-immune-profiling. The libraries resulting from the experiments were sequenced on a NovaSeq 6000 using a NovaSeq S4 200 cycles 2020 v1.5 kit, targeting using read 28, 10, 10, and 90 cycles targeting 20,000, 30,000, or 6000 reads per cell for gene expression, barcoded antigen, or Ig libraries, respectively. Libraries were analyzed using cellranger multi per manufacturer recommendation. Sequence analysis identified a total of 239 antibodies based on target and non-target (control) antigen UMI counts.

For the latter set of donors, we followed our previously described single-cell RNA sequencing protocol.^26^ To provide a concise overview, PBMCs underwent staining with an antibody cocktail (anti-human CD3e, CD4, CD8a, and CD14 on APC-Cy7, CD19 on PerCP-Cy5.5), antigen baits (panel of the current circulating VoC trimerized S and HSA negative control) on Totalseq PE and APC/or non-fluorophore barcoded-streptavidin, and LIVE/DEAD Fixable Aqua Dead Cell Stain Kit (Thermo) to distinguish live antigen double-positive B cells via FACS. Subsequently, the sorted cells were centrifuged and resuspended into the appropriate volume recommended for the 10× Genomic 5′V2 Single Cell Immune Profiling kit. Sequencing and initial cellranger analysis of the resulting libraries was identical as previously described. After removing unproductive HC/LC paired clones and doublets, the assignment of antigen specificity was accomplished by establishing individual baseline thresholds at the 99.7th percentile for each antigen bait. This was achieved by examining the background noise level in “empty” droplets (those without detected cells) and creating a negative binomial distribution of UMI-normalized values for each antigen bait. Only cells surpassing the established baseline threshold were considered positive for the corresponding antigen.

#### Sequencing analysis

Sequencing data were analyzed and visualized using Cell Ranger, enclone, scab, and custom Python and R scripts. Scab is open source and freely available at github.com/briney/scab.

### QUANTIFICATION AND STATISTICAL ANALYSIS

Antibody neutralizing activity (IC_50_) was determined using the log(inhibitor) vs. response equation with the variable slope on the GraphPad Prism software. Phylogenetic analysis was performed by sequence alignment and computation of a pairwise sequence identity score between individual pairs of heavy chains. These scores were then utilized to construct a distance matrix, which was subsequently employed in a Neighbor-Joining clustering approach to group the sequences together. For the animal study, significance was determined using a one-way ANalysis Of VAriance (ANOVA) with Dunnet’s multiple comparisons test. We define statistical significance as a *p*-value less than 0.05.

## Supplementary Material

1

## Figures and Tables

**Table:** KEY RESOURCES TABLE

REAGENT or RESOURCE	SOURCE	IDENTIFIER
Antibodies		

TotalSeq^™^-C0251 anti-human Hashtag 1 Antibody	Biolegend	Cat# 394661; RRID: AB_2801031
TotalSeq^™^-C0252 anti-human Hashtag 2 Antibody	Biolegend	Cat# 394663; RRID: AB_2801032
TotalSeq^™^-C0253 anti-human Hashtag 3 Antibody	Biolegend	Cat# 394665; RRID: AB_2801033
TotalSeq^™^-C0254 anti-human Hashtag 4 Antibody	Biolegend	Cat# 394667; RRID: AB_2801034
TotalSeq^™^-C0255 anti-human Hashtag 5 Antibody	Biolegend	Cat# 394669; RRID: AB_2801035
TotalSeq^™^-C0256 anti-human Hashtag 6 Antibody	Biolegend	Cat# 394671; RRID: AB_2820042
BD Pharmingen^™^ APC-Cy^™^7 Mouse Anti-Human CD3	BD Biosciences	Cat# 557757; RRID: AB_396863
BD Pharmingen^™^ APC-Cy^™^7 Mouse Anti-Human CD8	BD Biosciences	Cat# 557760; RRID: AB_396865
BD Pharmingen^™^ APC-H7 Mouse Anti-Human CD14	BD Biosciences	Cat# 561384; RRID: AB_10611720
APC/Cyanine7 anti-human CD4 Antibody	Biolegend	Cat# 317418; RRID: AB_571947
PerCP/Cyanine5.5 Mouse Anti-Human CD19	Biolegend	Cat# 302230; RRID: AB_2073119
BD^™^ PE-Cy^™^7 Mouse Anti-Human CD19	BD Biosciences	Cat# 341093

Bacterial and virus strains		

DH5alpha Super Competent Cells	BioPioneer	Cat# GACC-96P

Biological samples		

Sera and PBMC samples from convalescent	UCSD Human Research	UCSD IRB#200236
COVID-19 donors, vaccinated donors, and COVID-19 recovered-vaccinated donors	Protection Program	

Chemicals, peptides, and recombinant proteins		

Biotinylated Human Serum Albumin, His, Avitag	Acro Biosystems	Cat# HAS-H82E3
Biotinylated SARS-CoV-2 S protein (HV69–70del, Y144del, N501Y, A570D, D614G, P681H, T716I, S982A, D1118H) trimer, His, Avitag (Alpha)	Acro Biosystems	Cat# SPN-C82E5
Biotinylated SARS-CoV-2 Spike Trimer Protein (T19R, V70F, FR157–158Del, A222V, W258L, K417N, L452R, T478K, D614G, P681R, D950N), His, Avitag (Delta-plus)	Acro Biosystems	Cat# SPN-C82Ei
Biotinylated SARS-CoV-2 S protein, His, Avitag, Super stable trimer (WT)	Acro Biosystems	Cat# SPN-C82E9
Biotinylated SARS-CoV-2 S protein (L18F, D80A, D215G, 242–244del, R246I, K417N, E484K, N501Y, D614G, A701V) trimer, His, Avitag (Beta)	Acro Biosystems	Cat# SPN-C82E4
Biotinylated SARS-CoV-2 S protein (L18F, T20N, P26S, D138Y, R190S, K417T, E484K, N501Y, D614G, H655Y, T1027I, V1176F) trimer, His, Avitag (Gamma)	Acro Biosystems	Cat# SPN-C82E6
Biotinylated SARS-CoV-2 Spike Trimer Protein (T19R, G142D, EF156–157del, R158G, L452R, T478K, D614G, P681R, D950N), His, Avitag (Kappa)	Acro Biosystems	Cat# SPN-C82Ec
Biotinylated HCoV-HKU1 (isolate N5) Spike Trimer Protein, His,Avitag	Acro Biosystems	Cat# SPN-H82E3
Biotinylated HCoV-OC43 Spike Trimer Protein (RRSRG 754–758 GGSGG), His, Avitag	Acro Biosystems	Cat# SPN-H85E3
Human TruStain FcX	Biolegend	Cat# 422304
Biotinylated SARS-CoV2 Spike	Zhou et al.^19^	N/A
Biotinylated MERS-CoV Spike	Zhou et al.^19^	N/A
SARS-CoV-1 spike	Zhou et al.^19^	N/A
HCoV-OC43 spike	Zhou et al.^19^	N/A
FectoPRO	Polyplus	Cat# 116–001
Pierce^™^ IgG Elution Buffer	Thermo Fisher Scientific	Cat# 21004
LIVE/DEAD^™^ Fixable Aqua Dead Cell Stain Kit	Thermo Fisher Scientific	Cat# L34966
7-AAD (7-Aminoactinomycin D)	Thermo Fisher Scientific	Cat# A1310

Deposited data		

Local refinement of SARS-CoV-2 (HP-GSAS-Mut7) spike NTD in complex with TXG-0078 Fab	This study	PDB: 8SWH
Structure of SARS-CoV-2 (HP-GSAS-Mut7) spike in complex with TXG-0078 Fab -Conformation 1	This study	EMBD: 40812
Structure of SARS-CoV-2 (HP-GSAS-Mut7) spike in complex with TXG-0078 Fab -Conformation 2	This study	EMBD: 40813
Local refinement of SARS-CoV-2 (HP-GSAS-Mut7) spike NTD in complex with TXG-0078 Fab	This study	EMBD: 40814
Deep repertoire mining uncovers ultra-broad coronavirus neutralizing antibodies targeting multiple spike epitopes	This study	https://doi.org/10.5281/ZENODO.8432479

Experimental models: Cell lines		

Expi293F cells	Gibco	Cat# A14527
HeLa-ACE2	Zhou et al.^19^	N/A
FreeStyle293-F cells	Thermo Fisher Scientific	Cat# R79007
Vero E6 cells	ATCC	Cat# CRL-1586; RRID:CVCL_0574

Experimental models: Organisms/strains		

K18-hACE2 Mice	The Jackson Laboratory	Cat# 34860

Recombinant DNA		

pTwist CMV BG WPRE *Neo*	Twist Bioscience	N/A
pMDL	Addgene	Cat# 12251; RRID:Addgene_12251
pREV	Addgene	Cat# 12253; RRID:Addgene_12253
pVSV-G	Addgene	Cat# 8454; RRID:Addgene_8454
pCMV-dR8.2 dvpr	Addgene	Cat# 8455; RRID:Addgene_8455
pBOB-Luciferase	Addgene	Cat# 170674; RRID:Addgene_170674
phCMV3	Genlantis	Cat# P003300
pBOB-hACE2	Zhou et al.^19^	N/A
pBOB-Hdpp4	Zhou et al.^19^	N/A

Software and algorithms		

Cellranger v7.1.0	10X Genomics	N/A
Single Cell Analysis of B cells (SCAB) v0.2.1	Hurtado et al.^26^	N/A
Enclone v0.5.219	10X Genomics	N/A
Carterra’s Kinetics Tool software	Carterra	N/A
EPU 2 software	Thermo Fisher Scientific	N/A
ForteBio Data Analysis software	Sartorius	https://www.sartorius.com/en
cryoSPARC.v3	Punjani et al.^27^	N/A
Relion 3.1	Zivanov et al.^28^	N/A
Coot	Casañal et al., 2020^29^	N/A
Phenix	Liebschner, et al., 2019^30^	N/A
EMRinger	Barad et al., 2015^31^	N/A
MolProbity	Williams et al.^32^	N/A

Other		

Expi293 Expression Medium	Gibco	Cat# A1435101
Opti-MEM^™^	Thermo Fisher Scientific	Cat# 31985070
Tween 20	Sigma-Aldrich	Cat# P1379–500ML
Coming^®^ Dulbecco’s Phosphate-Buffered Saline, 1X without calcium and magnesium	Thermo Fisher Scientific	Cat# 21–031-CV
Fetal Bovine Serum	Omega Scientific, Inc.	Cat# FB-02
RPMI1640 medium	Thermo Fisher Scientific	Cat# 11875085
FreeStyle 293 Expression medium	Thermo Fisher Scientific	Cat# 12338018
Phusion^™^ High-Fidelity DNA Polymerase	Thermo Fisher Scientific	Cat# F-530XL
FACS tube with 70-μm mesh cap	Fisher Scientific	Cat# 08–771-23
SPRI beads	Beckman Coulter	Cat# B23318
Protein A Sepharose	GE Healthcare	Cat# 17096302
30K Amicon tubes	Millipore	Cat# UFC903024
Hamilton Microlab STAR platform	Hamilton Company	N/A
Carterra LSA SPR biosensor	Carterra	N/A
HC30M chip	Carterra	N/A
Octet Anti-human Fc capture (AHC) biosensors	ForteBio	Cat# 18–5063
Octet Streptavidin (SA) Biosensors	ForteBio	Cat# 18–5019
96-well half-area high binding plates	Corning	Cat# 3690
96-well plates	Corning	Cat# 3916
Quantifoil^®^ R 1.2/1.3 300 Mesh, Au	Quantifoil Micro Tools GmbH	Cat# Q3100AR1.3
Prometheus NT.48 NanoDSF	NanoTemper Technologies	N/A
Leica DMi1 Inverted Microscope	Leica Microsystems	N/A
Tecnai F20 electron microscope	FEI	N/A
TemCam F415 CMOS camera	TVIPS	N/A
Vitrobot mark IV	Thermo Fisher Scientific	N/A
Solarus 950 plasma system	Gatan	N/A
PELCO easiGlow	Ted Pella Inc.	N/A
Glacios	Thermo Fisher Scientific	N/A
Falcon 4 direct electron detector	Thermo Fisher Scientific	N/A
Chromium Next GEM Single Cell 5′ Kit v2, 16 rxns	10× Genomics	Cat# 1000263
5′ Feature Barcode Kit, 16 rxns	10× Genomics	Cat# 1000541
Library Construction Kit, 16 rxns	10× Genomics	Cat# 1000190
Chromium Next GEM Chip K Single Cell Kit, 16 rxns	10× Genomics	Cat# 1000287
Dual Index Kit TT Set A 96 rxns	10× Genomics	Cat# 1000215
Dual Index Kit TN Set A, 96 rxn	10× Genomics	Cat# 1000250
Chromium Single Cell Human BCR Amplification Kit, 16 rxns	10× Genomics	Cat# 1000253
Chromium Controller	10× Genomics	N/A
NovaSeq S4 200 cycles v1.5 kit	Illumina Inc.	Cat# 20028313
NovaSeq 6000 Sequencing System	Illumina Inc.	N/A
TotalSeq^™^-C0951 PE Streptavidin	Biolegend	Cat# 405261
TotalSeq^™^-C0952 PE Streptavidin	Biolegend	Cat# 405263
TotalSeq^™^-C0953 PE Streptavidin	Biolegend	Cat# 405265
TotalSeq^™^-C0954 PE Streptavidin	Biolegend	Cat# 405267
TotalSeq^™^-C0962 PE Streptavidin	Biolegend	Cat# 405153
TotalSeq^™^-C0956 APC Streptavidin	Biolegend	Cat# 405283
TotalSeq^™^-C0957 APC Streptavidin	Biolegend	Cat# 405285
TotalSeq^™^-C0958 APC Streptavidin	Biolegend	Cat# 405293
TotalSeq^™^-C0971 Streptavidin	Biolegend	Cat# 405271
TotalSeq^™^-C0972 Streptavidin	Biolegend	Cat# 405273
TotalSeq^™^-C0973 Streptavidin	Biolegend	Cat# 405277
FACSMelody Cell sorter	BD Biosciences	N/A

## Highlights

- Next-generation antigen barcoding allows deep mining of antigen-specific antibody repertories
- TXG-0078 achieves remarkable breadth by uniquely targeting the SARS-CoV-2 N-terminal domain
- A cocktail of complementary coronavirus-specific antibodies shows *in vivo* protection
