# Supplementary material for: Deep repertoire mining uncovers ultra-broad coronavirus neutralizing antibodies targeting multiple spike epitopes
Source: Cell Rep. Author manuscript; Available in PMC 2024 Dec 26. (PMC11671098; doi:10.1016/j.celrep.2024.114307)
Supplement: 1 [file NIHMS2005237-supplement-1.pdf]

Cell Reports, Volume *43*

Supplemental information

Deep repertoire mining uncovers ultra-broad coronavirus neutralizing antibodies targeting multiple spike epitopes

Jonathan Hurtado, Thomas F. Rogers, David B. Jaffe, Bruce A. Adams, Sandhya Bangaru, Elijah Garcia, Tazio Capozzola, Terrence Messmer, Pragati Sharma, Ge Song, Nathan Beutler, Wanting He, Katharina Dueker, Rami Musharrafieh, Sarah Burbach, Alina Truong, Michael J.T. Stubbington, Dennis R. Burton, Raiees Andrabi, Andrew B. Ward, Wyatt J. McDonnell, and Bryan Briney

Supplementary Information

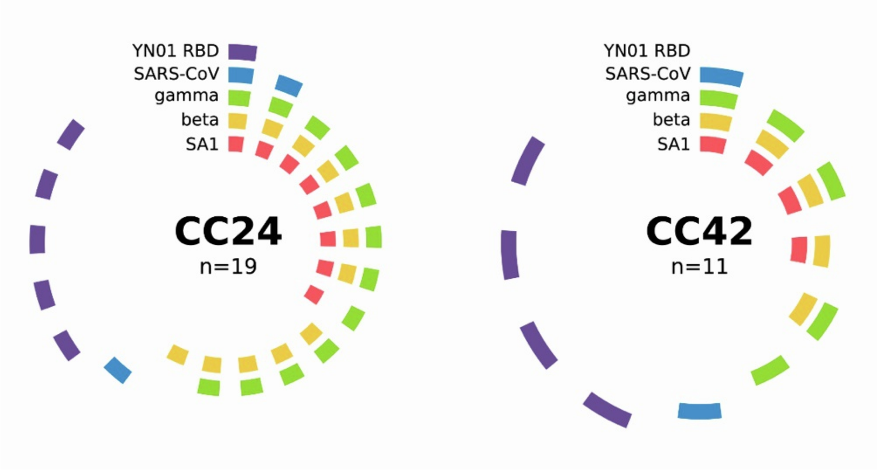

Figure S1. CoV-specific mAbs from two additional participants. Ring plots showing antigen barcode classifications for participants CC24 and CC42. Antigen rings are colored if the respective mAb was classified as antigen positive (WA1: magenta, beta: yellow, kappa: green, SARS-CoV: blue, YN02 RBD: purple).

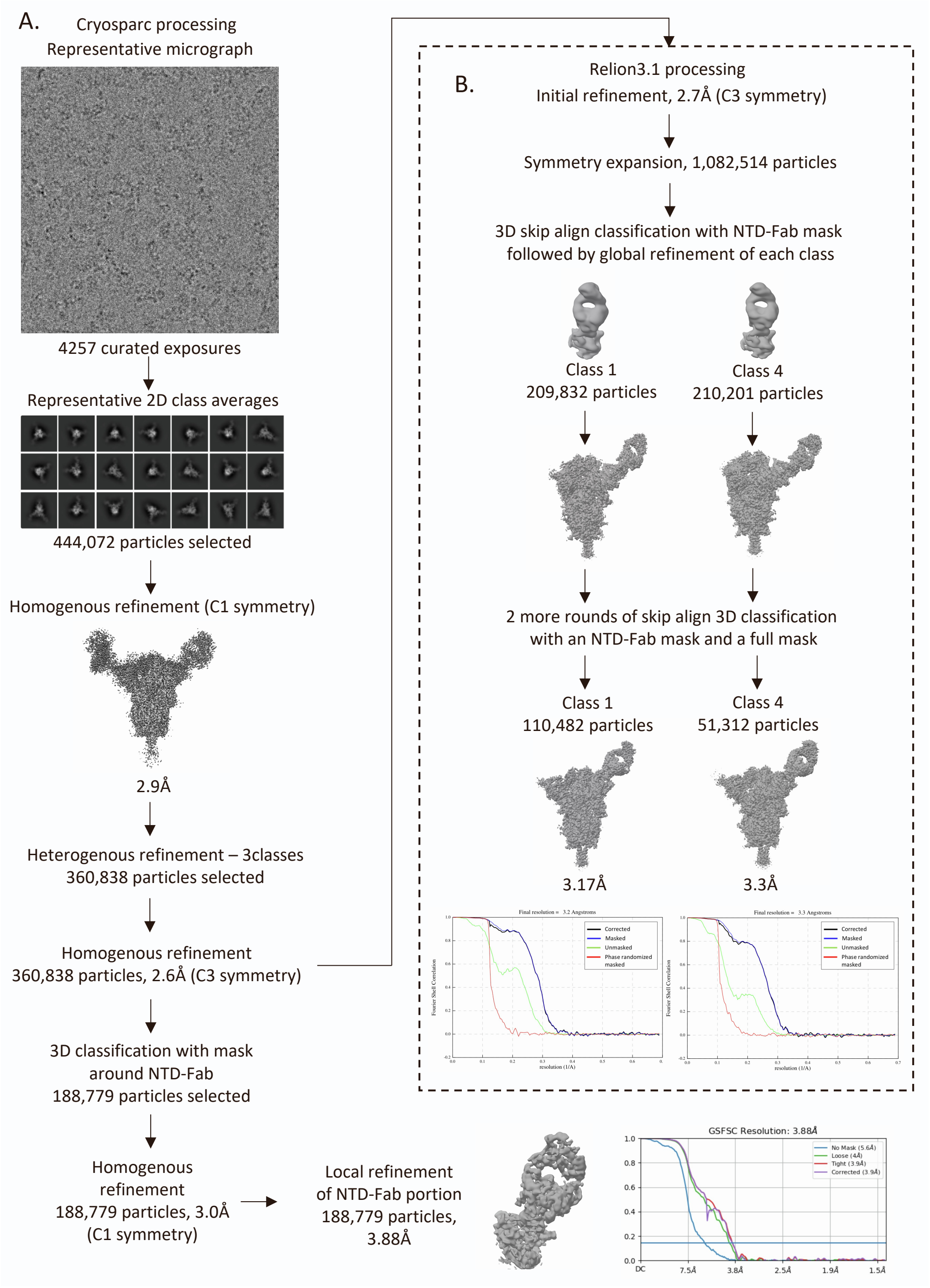

Figure S2. Schematic representation of the cryo-EM processing workflow for SARS-CoV-2 spike complexed with monoclonal Fab TXG-0078. **(A)** Workflow in cryoSPARC including initial processing steps (motion correction, CTF estimation, micrograph selection, particle picking, and selection based on 2D classification and heterogenous refinement) and local refinement of the spike NTD-Fab region used for model building. **(B)** Particle transfer to relion, symmetry expansion and iterative rounds of focused classification used to generate the two Fab-spike reconstructions revealing multiple states are shown in steps. The maps and corresponding FSC curves for each final reconstruction (C1 symmetry imposed) are provided in **(A)** and **(B).**

Table S1. CryoEM data collection, processing, and model building statistics.

**Map**	**SARS-CoV-2-HPM7-spike + TXG-0078-Fab Local refinement**	**SARS-CoV-2-HPM7-spike + TXG-0078-Fab conformation 1**	**SARS-CoV-2-HPM7-spike + TXG-0078-Fab conformation 2**
EMDB	EMD-xx	EMD-xx	EMD-xx
**Data collection**			
Microscope	TFS Glacios	TFS Glacios	TFS Glacios
Voltage (kV)	200	200	200
Detector	TFS Falcon 4	TFS Falcon 4	TFS Falcon 4
Recording mode	Counting	Counting	Counting
Nominal magnification	190,000x	190,000x	190,000x
Movie micrograph pixelsize (Å)	0.725	0.725	0.725
Number of frames (Falcon 4 EER fractions)	40	40	40
Total dose (e^-/Å^2)	50	50	50
Defocus range (µm)	-0.5 to -1.5	-0.5 to -1.5	-0.5 to -1.5
**EM data processing**			
Number of movie micrographs	4,257	4,257	4,257
Number of molecular projection images in map	188,799	110,482	51,312
Symmetry	C1	C1	C1
Map pixel size	0.725	0.725	0.725
Map resolution (FSC 0.143; Å)	3.88	3.17	3.3
Map sharpening B-factor (Å^2)	-149.6	-44.1	-36.5
**Structure building and validation**			
Number of residues in deposited model			
Amino acids	424	*n/a*	*n/a*
Carbohydrates	5	*n/a*	*n/a*
MolProbity score	1.1	*n/a*	*n/a*
Clashscore	1.65	*n/a*	*n/a*
EMRinger score	2.42	*n/a*	*n/a*
RMSD from ideal			
Bond length (Å)	0.021	*n/a*	*n/a*
Bond angles (°)	1.825	*n/a*	*n/a*
Ramachandran plot			
Favored (%)	96.83	*n/a*	*n/a*
Allowed (%)	3.17	*n/a*	*n/a*
Outliers (%)	0.00	*n/a*	*n/a*
Side chain rotamer outliers (%)	0.27	*n/a*	*n/a*
Cβ outliers (%)	0.00	*n/a*	*n/a*
PDB	xxxx	*n/a*	*n/a*
